# Nomadic Lactobacilli as cell factory for antibiofilm therapy

**DOI:** 10.3389/fcimb.2025.1668573

**Published:** 2025-10-16

**Authors:** Linette Shoby Paul, Hamitha Chinganadi Hameed, Leela K. V., Ashu Sharma, Jintae Lee, Moshe Shemesh, Satish Kumar Rajasekharan

**Affiliations:** ^1^ Department of Biotechnology, School of Bioengineering, SRM Institute of Science and Technology, Tamil Nadu, Kattankulathur, India; ^2^ Department of Microbiology, SRM Medical College Hospital and Research Centre (SRMMCH&RC), SRM Institute of Science and Technology, Kattankulathur, India; ^3^ Department of Oral Biology, School of Dental Medicine, University at Buffalo, Buffalo, NY, United States; ^4^ School of Chemical Engineering, Yeungnam University, Gyeongsan, Republic of Korea; ^5^ Department of Food Science, Institute of Postharvest Technology and Food Sciences, Agricultural Research Organization (ARO), Volcani Institute, Rishon LeZion, Israel

**Keywords:** cellular heterogeneity, V-shaped structure, probiotic biofilm, Lactobacilli, *C. albicans*, nomadic

## Abstract

Vulvovaginal candidiasis (VVC) is an infection caused by *Candida albicans* that presents an escalating threat to humans. Lactobacilli may play a critical role in maintaining microbiome balance in the gut and vagina as well as limiting fungal colonization, including *C. albicans.* Certain Lactobacilli, classified as nomadic groups is gaining immense popularity in antifungal defense due to its unique morphological adaptations. One significant adaptation is the V-shaped cell chaining observed under low pH conditions governed by the LuxS-mediated quorum-sensing system. This structural adaptation potentiates altered secondary metabolite secretion. These geometric forms are not solely survival responses but reflect a structurally coordinated strategy that enhances both antibiofilm and antihyphal activities. In this perspective, we argue that morphology-driven transitions identify nomadic Lactobacilli as a promising frontier in probiotic therapy. By shifting from conventional probiotic formulations to structured microbial interventions, we propose the development of novel sustainable therapeutics for anticandidal therapy.

## 1 Etiology of vulvovaginal candidiasis

Vulvovaginal candidiasis (VVC) is a common and recurrent infection despite the availability of antifungal therapies. One major reason is the ability of *Candida albicans* to form biofilms, which enhances its resistance to treatment and immune elimination (Intra et al., 2022). Fluconazole is widely used, but reduced efficacy against biofilms is a major concern (Intra et al., 2022). While newer antifungals like ibrexafungerp and oteseconazole are alternatives, their long-term activity against biofilm-driven infections is still under evaluation (Intra et al., 2022). Misdiagnosis and frequent use of non-prescription antifungals lead to a delay in proper treatment, leading to recurrent episodes (Duar et al., 2017). These disadvantages raise an important question: can we develop simpler strategies to improve current therapies?

## 2 Nomadic Lactobacilli to tackle VVC

**Figure 1:**
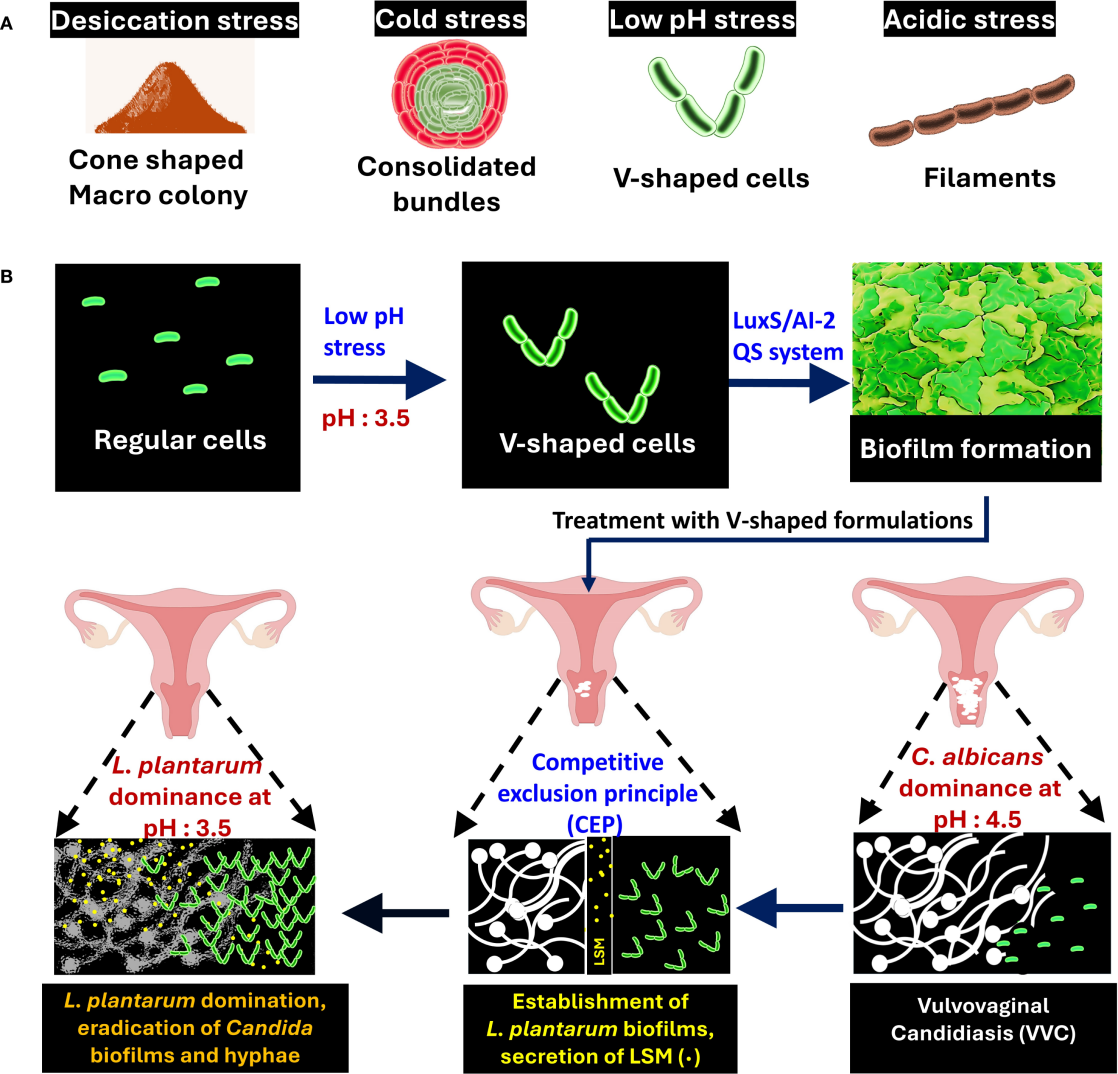
Morphological adaptation and protective role of *Lactiplantibacillus plantarum* in fighting *Candida albicans* biofilms in the vaginal microbiome. **(A)** Multicellular structures of *L. plantarum* formed under different stress conditions: desiccation (cone-shaped colonies), cold (bundles), low pH (V-shaped cells), and acidic stress (filaments). **(B)** *L. plantarum* transitions from regular cells to V-shaped cells under low pH stress, leading to biofilm formation via the LuxS/AI-2 Quorum Sensing (QS) system. Treatment with V-shaped formulations results in *L. plantarum* dominance, the competitive exclusion of *Candida* species, and the subsequent alleviation of Vulvovaginal Candidiasis (VVC).

Lactobacilli belong to a genus of Gram-positive, rod-shaped lactic acid bacteria that actively compete with *C. albicans* in the vaginal microbiome (Intra et al., 2022). These bacteria are broadly classified into free-living, host-adapted, and nomadic variants (Duar et al., 2017). Among these, certain species, particularly the nomadic variants (*Lactiplantibacillus plantarum*, *Lacticaseibacillus rhamnosus*, and *Lacticaseibacillus casei*), seem promising for antibiofilm therapy (Rajasekharan and Shemesh, 2022). The nomadic lifestyle of Lactobacillii refers to their ability to survive and adapt across different environments, rather than being localized to a singular habitat. These bacteria can shift between various environmental habitats such as soil, plant surfaces, aquatic environments, fermented foods, the oral cavity and the gastrointestinal tract (Duar et al., 2017). They maintain their genomic and metabolic flexibility that allows them to reshape their phenotype according to the habitat. The current thinking in the field is that the geometrical structuring facilitates the nomadic lifestyle, leading to successful adaptation of Lactobacilli to different environmental niches, though the mechanistic understanding of structural changes enabling their nomadic lifestyle remains an active area of research*. L. plantarum* is one such nomadic variant that was recently shown to demonstrate multifaceted cell structures such as cone-shaped colonies, sacrifice-for-survival bundles, and V-shaped cell chaining in response to acidic pH conditions (**Figure 1a**) (Rajasekharan and Shemesh, 2022; Venugopal et al., 2025). Findings reveal that in acidic environment, mimicking the vaginal niche, *L. plantarum* transitions are linked to LuxS quorum-sensing pathways (Venugopal et al., 2025). This structural state coincides with modified metabolite secretion and raises questions about whether morphology-driven metabolic shifts enhance its antifungal capacity. This is particularly relevant in the context of VVC, a common mucosal infection affecting up to 75% of women during their reproductive years (Sobel, 2007; Intra et al., 2022). *C. albicans*, the primary causative agent, often forms drug-resistant biofilms and evades host immunity (Zeise et al., 2021; Poon and Hui, 2023). While conventional antifungals face increasing resistance, probiotics, particularly those containing Lactobacilli, offer an emerging alternative by restoring microbial balance, inhibiting *Candida* adhesion, biofilm formation and modulating immune responses (**Table 1**). Clinical studies have shown that species like *L. plantarum* underscoring their therapeutic value (Bertarello et al., 2024). We propose that structural shifts in *L. plantarum*, especially the formation of V-shaped structures alleviate symptoms and reduce recurrence in VVC-infected cells, which may be a functional adaptation for enhanced antifungal activity. This explores how the morphology-metabolite interplay could influence the next generation of probiotic design to target *Candida* biofilms and overcome antifungal resistance.

**Table 1:** Comparative analysis of antifungal properties of Lactobacilli against *Candida albicans* biofilms.

Lactobacilli	*Candida* species targeted	Antifungal effect	Mechanism of action	Additional effects	References
LP 8014 CFS	*C. albicans* SC5314	Reduced biomass & metabolic activity of biofilm	Biofilm inhibition & filamentation suppression via Lactobacilli-secreted metabolites	Metabolite-mediated suppression	(Intra et al., 2022)
*L. rhamnosus* GG CFS	*C. albicans A14*	Reduced number of filaments (prevents yeast-hyphae transition)	Downregulation of ALS1, ALS3, EFG1, TEC1 pH-independent activity	Metabolic competition	(Intra et al., 2022)
Gasseri *L. crispatus*	*C. albicans*	Inhibition of biofilm formation & reduction of fungal adhesion	Secretion of antifungal metabolites	None	(Duar et al., 2017)
*L. fermentum* SNUV175 *L. crispatus* SNUV220	*C. albicans*	Inhibition of hyphal growth	Secretion of heat-stable, non-proteinaceous antifungal compounds	None	(Jang et al., 2019)
*L. rhamnosus*	*C. albicans*	Reduction in population	Nutrient depletion, metabolic stress induction & filamentation inhibition	Metabolic environment modulation	(Alonso-Roman et al., 2022)
SD5870 CBS N116411 DSM 14658	*C. albicans*	Prevention of biofilm formation	Not fully elucidated	None	(James et al., 2016)
*L. rhamnosus* GR-1 *L. reuteri* RC-14	*C. albicans*	Growth inhibition, biofilm suppression, and reduction in *C. albicans* population	pH reduction, gene expression modulation, nutrient competition, and secretion of antifungal metabolites	Adhesion interference	(Köhler et al., 2012)
BC1-BS LP-BS HY-LP-BS	*C. albicans*	Reduction in biofilm formation	Prevents adhesion & destabilizes biofilm matrix via biosurfactant action	None	(Abruzzo et al., 2021)
*L. rhamnosus* GG	*C. albicans*	Hyphal growth inhibition & adhesion blocking	Competitive exclusion, glucose depletion, and hyphal gene downregulation	None	(Mailänder-Sánchez et al., 2017)
*L. casei* / pPG 612.1-BLF	*C. albicans*	Growth suppression & BLF-mediated antifungal activity	Iron Sequestration, Direct Antimicrobial Activity and Immunomodulation	None	(Liao et al., 2019)
*L. crispatus*	*C. albicans*	Growth inhibition & suppresses fungal proliferation	pH Reduction, Secretion of Inhibitory Compounds and Impairment of Fungal Adhesion	None	(Patil et al., 2015)
*L. pentosus* KCA1 *L. plantarum* WCFS1 *L. rhamnosus* GG	*C. albicans*	Hyphal inhibition	no specific mechanism	None	(Oerlemans et al., 2020)
*L. rhamnosus*	*C. albicans*	Blocks yeast-hyphae transition	1-ABC secretion and Kinase inhibition (Yak1)	Prevention of invasive structure formation	(MacAlpine et al., 2021)
*L. crispatus*	*C. albicans*	Growth inhibition	Lactic Acid Production, Nutrient Competition, Hydrogen Peroxide Production and Host Cell Adhesion	None	(Patil et al., 2015)
*L. crispatus* SNUV220 & *L. fermentus* SNUV176	*C. albicans*	Growth inhibition	Secretion of pH-independent antifungal compounds	None	(Zeise et al., 2021)
V Shapes	*C. albicans*	Biofilm growth suppression	V-shaped cell chaining and Biofilm formation	None	(Rajasekharan and Shemesh, 2022)

## 3 The V-shaped structuring

Biofilm formation is a key survival strategy for microbes in mucosal environments. In the vaginal niche, *L. plantarum* typically dominates through acidification and spatial exclusion, but not all species are equally effective against *C. albicans*. The environment provides signals such as acid stress, bile salts, osmotic stress, temperature fluctuations and nutrient availability that can have a direct impact on the morphology of Lactobacilli. The V-shaped structuring can be formed predominantly in the vaginal environment, where relatively high acidity may facilitate such cellular transformation. The structured cells possess enhanced biochemical defense mechanisms that effectively inhibit hyphal extension and biofilms, as was shown in a *C. elegans* model (Rajasekharan and Shemesh, 2022). This V-shaped structuring correlates with increased surface adherence and more cohesive biofilm formation compared to other known shapes, allowing it to physically outcompete *C. albicans* establishment (Rajasekharan and Shemesh, 2022). (**Figure 1b**). In our experimental models, V-shaped *L. plantarum* cells effectively inhibited yeast virulence in *in vitro* and *in vivo* nematode models (Rajasekharan and Shemesh, 2022). This suggests that the V-shaped morphology is not a passive stress response, but rather an adaptive strategy for enhanced mucosal colonization and spatial exclusion of fungal pathogens. The role of *Candida* in shaping Lactobacilli morphology remains unclear, but it is conceivable that the dysbiosis caused by the colonization of *Candida* may indeed be related to its possible impact on Lactobacilli morphology. Further studies are warranted to elucidate possible role during microbial competition and antagonistic interactions. **Figure 1b** illustrates how the geometrical structuring may lead to physical suppression of *Candida* colonization through dense probiotic biofilms.

## 4 Morphotype-driven regulation of Lactobacilli-secreted metabolites

**Figure 2:**
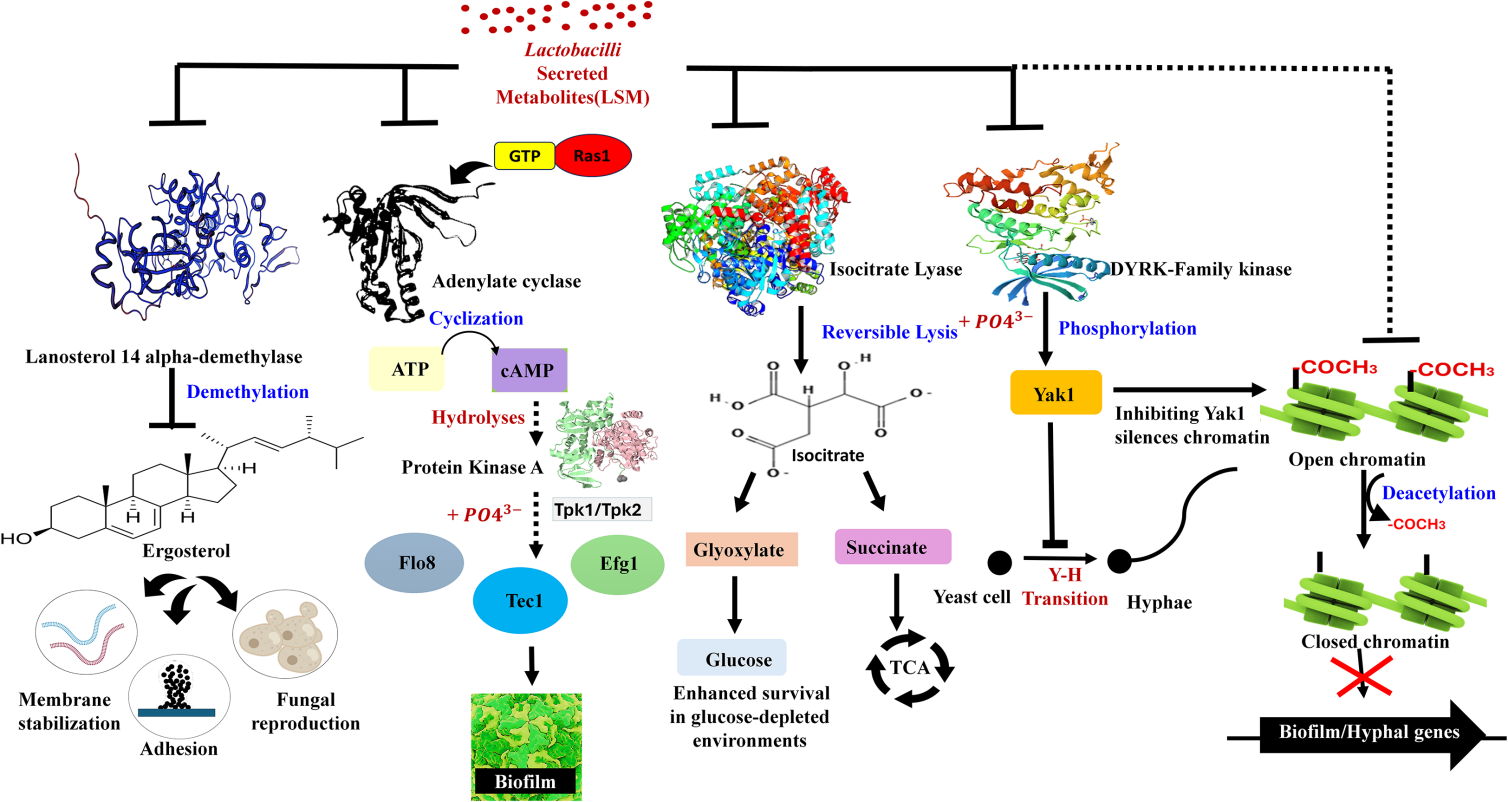
Inhibitory mechanisms of Lactobacilli*-*secreted metabolites (LSMs) on biofilm and hyphal regulatory pathways in *Candida albicans.* The schematic diagram illustrates the plausible pathways by which LSM might inhibit fungal pathogenesis (biofilm and hyphae). These pathways include Ras1/cAMP signaling, ergosterol biosynthesis, glyoxylate metabolism, DYRK/Yak1 and chromatin regulation.

Although numerous antimicrobial compounds of Lactobacilli are well established, how they relate to structural morphology remains unexplored. Based on the results discussed in Section 3, we found that *L. plantarum* under acidic stress exhibits a distinct metabolic shift associated with the previously described chaining morphology. Notably, this morphotype displayed increased secretion of metabolites of cyclic dipeptides, which are stable and interfere with the maturation of biofilms (Narasimulu et al., 2024). These compounds were more abundant in the V-shaped state compared to rod-shaped cells, indicating that the structural adaptation also enhances metabolite secretion. This observation proposes a novel concept in probiotic design; morphological states may directly influence the potency and spectrum of antimicrobial metabolites. Notably, formulated V-shaped cells hold significant potential to be made into probiotic products, thus merging the structural advantages with practical formulating benefits. Such structure-informed probiotic strategies could yield targeted solutions for recurrent *Candida* infections. In the following section, we detail the intracellular targets of these metabolites and how they plausibly disrupt *C. albicans* biofilm formation (**Figure 2**).

## 5 Biofilm-based targets of Lactobacilli-secreted metabolites

We examine the proposed role of morphology-driven metabolite adaptation on how Lactobacilli-secreted metabolites (LSMs) interfere with intracellular pathways that regulate the formation and survival of *C. albicans* biofilms. Several LSMs have been shown to inhibit adenylate cyclase, which can disrupt the RAS-cAMP-MAPK signaling cascade and downstream regulators such as *tpk1, efg1, flo8*, and *tec1*, which are crucial transcription factors that drive hyphal transition and biofilm maturation (Alonso-Roman et al., 2022). LSMs, such as pyruvate and oxaloacetate, may interfere with metabolism for competitive inhibition of isocitrate lyase, which inhibits the glyoxylate cycle and *C. albicans* ability to grow in nutrient-poor mucosal environments. Other targets include lanosterol 14α-demethylase, which disrupts ergosterol synthesis and destabilizes fungal membranes. Compounds like 1-acetyl-β-carboline (1-ABC) directly inhibit Yak1, a kinase critical for morphogenesis and biofilm development (MacAlpine et al., 2021). Additionally, sodium butyrate, a short-chain fatty acid and histone deacetylase inhibitor (HDACi), impairs fungal gene expression by altering chromatin organization and structure (Poon and Hui, 2023). These proposed mechanisms are illustrated in **Figure 2**, which integrates our hypothesis with known fungal regulatory pathways. Increased biofilm inhibitory activity of V-shaped *L. plantarum* may not only come from competitive exclusion but also from a specialized LSM profile that precisely targets fungal virulence factors. Understanding these molecular interactions will open a path toward next-generation probiotic therapies for persistent biofilm-associated infections such as recurrent VVC.

## 6 Concluding remarks and future perspectives

Our investigation indicates that structural adaptation of *L. plantarum*, more especially, its transition to V-shaped cells in vaginal-like acidic environments, may have a major impact on the synthesis of antifungal metabolites. This presents an intriguing hypothesis that cell morphology is a functional state that improves probiotic competitiveness against *C. albicans.* A promising bioactive profile for preventing biofilm-associated infections might be attributed to cyclic dipeptides and other specialized LSMs, including Prolyl-arginine, Valyl-threonine, and Valyl-glutamine (Narasimulu et al., 2024). Future research should focus on characterizing these metabolic pathways and understanding how they interface with fungal signaling and host immunity. Unlocking the regulatory controls behind such transitions could lead to novel bioengineered probiotic therapies designed not just by strain selection, but by regulating growth conditions and structure to maximize antifungal efficacy. This structure-guided approach may open a new path to address drug resistance and design more robust microbiome-based interventions for Biofilm-associated infection. While our research highlights the structural and metabolic role of *L. plantarum*, other nomadic Lactobacilli such as *L. rhamnosus* and *L. casei* remain largely unexplored and warrants further studies.

## Data Availability

The original contributions presented in the study are included in the article/supplementary material. Further inquiries can be directed to the corresponding authors.
